# The complete chloroplast genome of *Phlomoides younghushandii* (Lamiaceae), a traditional Tibetan medicinal herb

**DOI:** 10.1080/23802359.2021.1902413

**Published:** 2021-03-24

**Authors:** Dao-Zhang Min, Fei Zhao, Qiong Zhang, Bo Li

**Affiliations:** a Research Centre of Ecological Sciences, College of Agronomy, Jiangxi Agricultural University, Nanchang, PR China; b CAS Key Laboratory for Plant Diversity and Biogeography of East Asia, Kunming Institute of Botany, Chinese Academy of Sciences, Kunming, PR China

**Keywords:** Chinese herbal medicine, *Lamiodide*, *Phlomideae*, *Phlomoides*, plastome

## Abstract

The species *Phlomoides younghushandii* is a medicinal herb mainly distributed in southwest China. The first complete plastid genome sequence of *P. younghushandii* reported here was 151,747 bp long, with the large single copy (LSC) region of 83,181 bp, the small single copy (SSC) region of 17,372 bp and two inverted repeats (IRa and IRb) of 25,597 bp. The plastome contained 114 genes, including 80 protein-coding genes, four encoding rRNAs, and 30 encoding tRNAs. The overall GC content was 38.5%. Phylogenetic analysis of Lamiaceae based on a whole plastome matrix suggested that *Phlomoides* is closely related to the genus *Phlomis* as members of subfamily Lamioideae.

As currently defined (Scheen et al. 2010; Bendiksby et al. 2011; Salmaki et al. 2012), Phlomideae consists of two genera, *Phlomis* L. (50–90 spp.) and *Phlomoides* Moench (150–170 spp.). The latter genus is highly diverse in the Iranian highlands, Central Asia, and China (Khosroshahi and Salmaki 2019; Zhao et al. 2019). *Phlomoides younghushandii* (S.M. Mukerjee) Kamelin & Makhm. is a perennial herb, widely distributed in the Qinghai–Tibet Plateau (QTP), and is commonly used in traditional Chinese medicine (TCM) to treat colds, sore ulceration, bronchitis, and other diseases (Peng 2017).

Young leaves of *P. younghushandii* were freshly collected in Langkazi County, Xizang, China (90°24′33.61″E, 29°06′10.72″N) and dried immediately in silica gel. A voucher specimen was deposited in the Herbarium of Kunming Institute of Botany, Chinese Academy of Sciences (Herbarium Code: KUN, URL: http://kun.kingdonia.org/, contact Prof. Zhu-Liang Yang *via* fungi@mail.kib.ac.cn) under the voucher number *Chen et al*. *EM1033*. Total genomic DNA was isolated using the CTAB method (Doyle and Doyle 1987) and fragmented into ca. 300 bp size by transposome tagmentation using the Nextera XT kit. A paired-end library was constructed according to the manufacture’s protocol (NEBNext^®^ Ultra II^™^DNA Library Prep Kit) and sequenced on the Illumina HiSeq 2000 using the 150 PE kit (BGI-Shenzhen, Guangdong, China).

Illumina paired-end sequencing produced 13,640,496 raw reads for the *P. younghushandii.* The high-quality clean reads were carried out using Fast QC toolkit (Andrews 2010) with the parameter set as Q ≥ 25. *De novo* assembly was conducted with the GetOrganelle pipeline (https://github.com/Kinggerm/GetOrganelle, Jin et al. 2020) under the optimal k-mer 105, and the resulting plastome was annotated in Geneious version 11.0.3 (Kearse et al. 2012) using the previously published *Phlomoides betonicoides* (Diels) Kamelin & Makhm plastome as reference (Zhao et al. 2019). The annotated plastid genome sequence was deposited on GenBank with accession number MW405448.

The whole plastid genome of *P*. *younghushandii* was 151,747 bp, with a large single-copy (LSC) region (83,181 bp), a small single-copy (SSC) region (17,372 bp), and a pair of inverted repeats (IRa and IRb: 25,597 bp). The annotated genome comprised 114 genes, including 80 protein-coding genes, four ribosomal RNA genes (*rrn16*, *rrn23*, *rrn4.5*, and *rrn5*), and 30 transfer *RNA* genes.

Eighteen genes were duplicated in the IR regions, including seven protein-coding genes (*ndhB*, *rpl2*, *rpl23*, *rps12*, *rps7*, *ycf2*, and *ycf15*), four ribosomal *RNA* genes (*rrn16*, *rrn23*, *rrn4*.5, and *rrn5*), and seven transfer *RNA* genes (*trnA*-^UGC^, *trnI*-^CAU^, *trnI*-^GAU^, *trnL*-^CAA^, *trnN*-^GUU^, *trnR*-^ACG^, and *trnV*-^GAC^). The overall GC content of *P*. *younghushandii* plastid genome is 38.5% (LSC, 36.7%; SSC, 32.6%; IRs, 43.4%).

Maximum-likelihood (ML) phylogenetic analysis was conducted using RAxML version 8.1.11 (Stamatakis 2014) as implemented on the Cyberinfrastructure for Phylogenetic Research (CIPRES) Science Gateway (http://www.phylo.org/, Miller et al. 2010), employing the GTR + G model with 1000 bootstrap iterations (-#j-N). Other parameters used the default settings. Phylogenetic analysis based on 80 protein-coding genes of 38 representative plastomes within the family Lamiaceae further confirmed that *Phlomoides* is a member of the subfamily Lamioideae (Figure 1).

**Figure 1.**
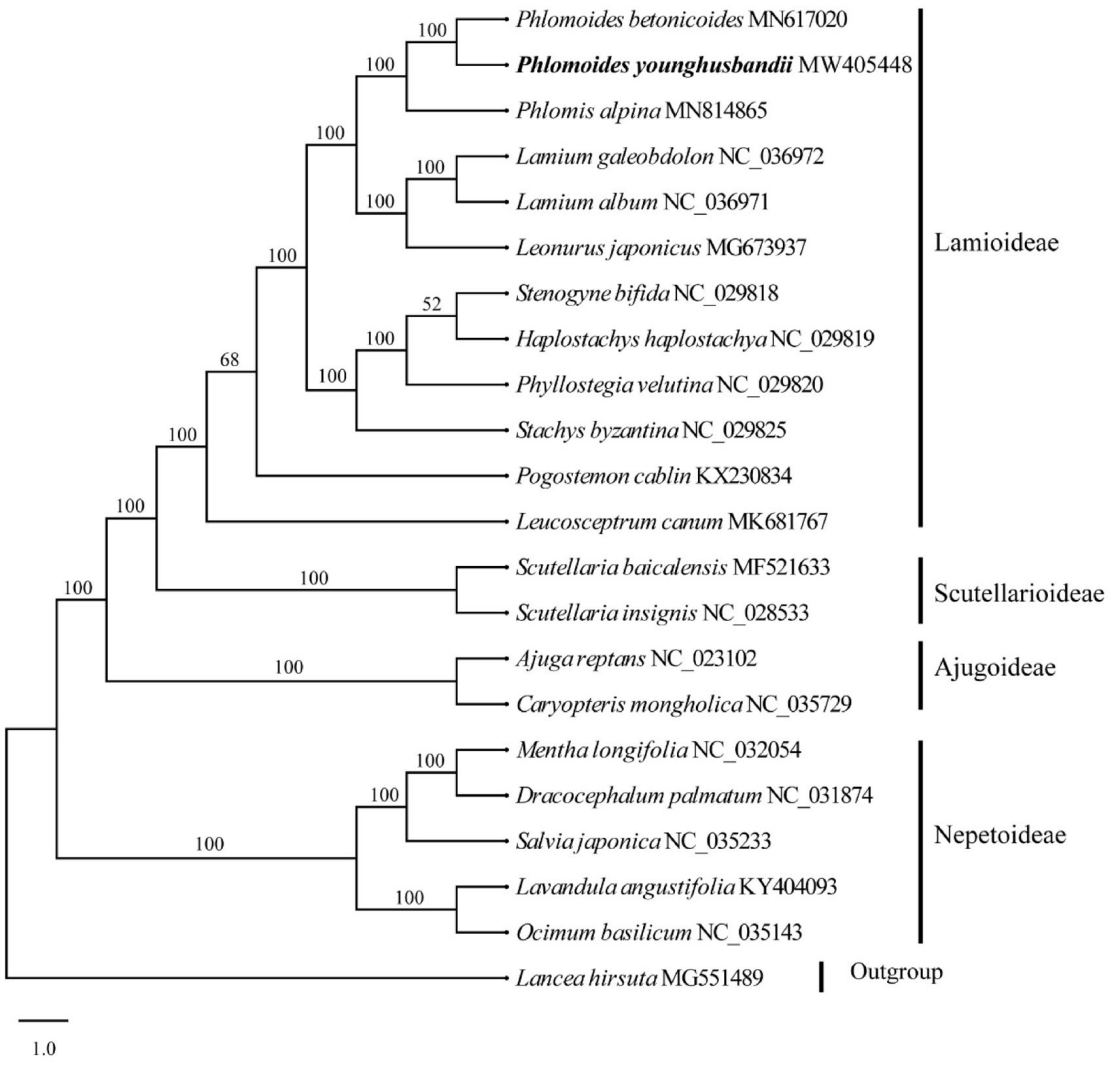
Maximum likelihood (ML) tree of Lamiaceae inferred from 80 protein-coding genes of 25 plastomes (including the outgroup *Lancea hirsuta*). Bootstrap support values are indicated on branches.

## Data Availability

The data that support the findings of this study are openly available in GenBank of NCBI at https://www.ncbi.nlm.nih.gov under the accession no. MW405448, and the raw sequenced reads was submitted to the Sequence Read Archive (SRA) database under the Bioproject number PRJNA687635.
